# Registration of polarimetric images for *in vivo* skin diagnostics

**DOI:** 10.1117/1.JBO.27.9.096001

**Published:** 2022-08-30

**Authors:** Lennart Jütte, Gaurav Sharma, Harshkumar Patel, Bernhard Roth

**Affiliations:** a Leibniz University Hannover, Hannover Centre for Optical Technologies, Hannover, Germany; b Leibniz University Hannover, Cluster of Excellence PhoenixD, Hannover, Germany

**Keywords:** Mueller matrix, biomedical imaging, polarimetry, dermoscopy, image registration

## Abstract

**Significance:**

Mueller matrix (MM) polarimetry is a promising tool for the detection of skin cancer. Polarimetric *in vivo* measurements often suffer from misalignment of the polarimetric images due to motion, which can lead to false results.

**Aim:**

We aim to provide an easy-to-implement polarimetric image data registration method to ensure proper image alignment.

**Approach:**

A feature-based image registration is implemented for an MM polarimeter for phantom and *in vivo* human skin measurements.

**Results:**

We show that the keypoint-based registration of polarimetric images is necessary for *in vivo* skin polarimetry to ensure reliable results. Further, we deliver an efficient semiautomated method for the registration of polarimetric images.

**Conclusions:**

Image registration for *in vivo* polarimetry of human skin is required for improved diagnostics and can be efficiently enhanced with a keypoint-based approach.

## 1 Introduction

Imaging of the human skin with polarimetric techniques has gained importance in recent years, as the incidence of melanoma, which is the deadliest type of skin cancer, is rising throughout all skin types.^1^ Beyond the classical skin cancer screening done by a dermatologist with a dermoscope,^2^ new modalities are being implemented for the early detection of melanoma: polarimetric imaging devices such as Stokes^3^ or Mueller polarimeters^4^^,^^5^ and machine learning-based computer-aided diagnosis systems^6^ are among the recently reported approaches. Furthermore, the early detection of melanoma is increasingly profiting from automated registration and analysis.^7^ In addition, adaptive optics such as adjustable liquid lenses allow for the implementation of autofocus in imaging systems without moving parts.^8^ However, because the up to 36 images necessary for Mueller matrix (MM) determination^9^ are typically obtained in acquisition times of 20 to 30 s, depending on the type of the setup,^10^ unintended body movements of the patient can become a significant limitation. Patient motion can lead to misalignment of the polarimetric images.^11^ Further, it can induce motion blur, in particular because laser power levels have to be reduced due to laser safety and therefore relatively high exposure times are needed to acquire the MMs. In addition, the motion can add up in cases in which a summation process of the polarimetric data is needed to enhance the signal-to-noise ratio (SNR).^11^ For example, the registration of scanned polarimetric images for ophthalmology was investigated by Nourrit et al.^11^ The above-mentioned problems in the MM acquisition can be partly overcome by hardware upgrades, e.g., camera and lens upgrades. However, such upgrades typically rely on complex and costly components.^12^ Therefore, software solutions represent an alternative approach and promise improved results.

In this work, we investigate the need and potential benefits of registering polarimetric images for application *in vivo* for dermoscopy. Registration of such data is particularly complex because the skin rarely shows sufficient key points with strong contrast (except for mole evaluation^13^). In addition, the contrast of key points usually changes considerably between the polarization states of the lighting and the analyzer. This issue and the possibility of key points moving out of the field of view make it difficult to detect the same set of key points within the up to 36 images typically acquired in MM polarimetry (MMP). To evaluate the potential benefits of this method, we determine the MM and its polarimetric parameters with and without the polarimetric image registration for the case of a printed melanoma phantom, healthy skin with a drop of honey, and a benign nevus, respectively. The instrumentation and registration schemes are described in Sec. 2. The results of registration and polarimetric analysis are presented in Sec. 3. Section 4 concludes the study.

## 2 Method

### 2.1 Instrumentation and Experimental Procedure

With MMP, the information about the polarization changing properties of a sample can be obtained.^14^ The MM combines the complete polarization properties of a sample within a 4×4 matrix.^15^ The sample is illuminated at different polarization states. The MM can be calculated from the intensity of the reflected or transmitted light.^16^ For *in vivo* skin imaging, only the reflection modality is relevant. Mathematically, the MM is a transformation matrix for Stokes vectors that describe the polarization state of light. The polarization state of the light after interaction with a sample $S_{0}$ is calculated from the Stokes vector of the incident light $S_{i}$ and the MM entries $M_{ij}$. of the sample as^17^

$$(\begin{array}{c} S_{o1}\\ S_{o2}\\ S_{o3}\\ S_{o4} \end{array})=[\begin{array}{cccc} M_{11} & M_{12} & M_{13} & M_{14}\\ M_{21} & M_{22} & M_{23} & M_{24}\\ M_{31} & M_{32} & M_{33} & M_{34}\\ M_{41} & M_{42} & M_{43} & M_{44} \end{array}](\begin{array}{c} S_{i1}\\ S_{i2}\\ S_{i3}\\ S_{i4} \end{array}).$$ (1)

These polarization states are described using Stokes vectors^18^ as follows:

$${\overset{\to }{S}}_{\text{Stokes}}=(\begin{array}{c} I_{H}+I_{V}\\ I_{H}-I_{V}\\ I_{P}-I_{M}\\ I_{R}+I_{L} \end{array})=(\begin{array}{c} I_{H}+I_{V}\\ I_{H}-I_{V}\\ 2I_{P}-(I_{H}+I_{V})\\ 2I_{R}-(I_{H}+I_{V}) \end{array}).$$ (2)

The indices of the intensity values I stand for the polarization states as shown in Table 1.

**Table 1:** Explanation of indices and the correlated polarization states.

Index	H	V	P	M	R	L
Polarization	Horizontal	Vertical	Linear +45 deg	Linear −45 deg	Right circular	Left circular

The Stokes vector changes after the interaction of incident light with the sample, which is recorded to measure the MM. For the used setup, either 16 (needed states: H, V, P, and R) or 36 (needed states: H, V, P, M, R, and L) different images are acquired.^8^ Although the acquisition of the MM based on 16 images is faster and therefore results in less motion error, the usage of 36 images improves the SNR. More detailed descriptions of the formalism can be found in the literature.^17^ To reduce calibration measurement errors, in this work, we use six polarization states for the acquisition of the MM from the required 36 intensity measurements to increase the measurement accuracy and the SNR in comparison with the calculation of the MM from 16 images only.^19^ The calibration of the polarimeter is tested by measuring samples of known MMs such as commercially available polarizers, retarders, and diffusors as well as air. In general, the calibration of our polarimetric device involves two main steps. First, the relative orientation of the optical elements (i.e., linear polarizers and liquid crystal retarders) is set. Second, the physical behavior of the optical elements (i.e., the phase-shift of the liquid crystal retarders) needs to be calibrated by employing quarter- and half-wave-plates. The detailed calibration steps can be found in the literature.^20^ The results of the calibration procedures performed in this work are very consistent with the expected matrices. An increase in measurement time to improve the SNR needs to be carefully considered, especially *in vivo* measurements in which movement disorder can change the result.

The experimentally obtained MM entries do not show a direct relation to physical properties of the sample. Therefore, for a more detailed interpretation of the measurements, a polar decomposition is usually carried out. In this work, we use the common polar decomposition of Lu and Chipman^21^ in the pixel-by-pixel way with the rows of the pixel array indexed as i and the columns indexed as j:

$$M_{\exp,ij}\;=\;M_{\Delta ,ij}\cdot M_{R,ij}\cdot M_{D,ij}.$$ (3)

In this decomposition $M_{\exp}$ is the experimentally obtained MM, and $M_{\Delta }$, $M_{R}$, and $M_{D}$ represent the pure depolarizer, retarder, and diattenuator properties, respectively.^21^

The key parameters resulting from the polar decomposition are Δ, the depolarization power; R, the total retardance; D, the diattenuation; and P, the total polarizance. These physical properties of the sample are calculated from the following equations:^21^

$$\Delta_{ij}=1-\frac{\left|M_{22,ij}|+|M_{33,ij}|+|M_{44,ij}\right|}{3},$$ (4)

$$R_{ij}=\cos^{-1}(\frac{tr(M_{R,ij})}{2}-1),$$ (5)

$$P_{ij}=\frac{1}{M_{11,ij}}\sqrt{M_{21,ij}^{2}+M_{31,ij}^{2}+M_{41,ij}^{2}},$$ (6)

$$D_{ij}=\frac{1}{M_{11,ij}}\sqrt{M_{12,ij}^{2}+M_{13,ij}^{2}+M_{14,ij}^{2}}.$$ (7)

**Fig. 1:**
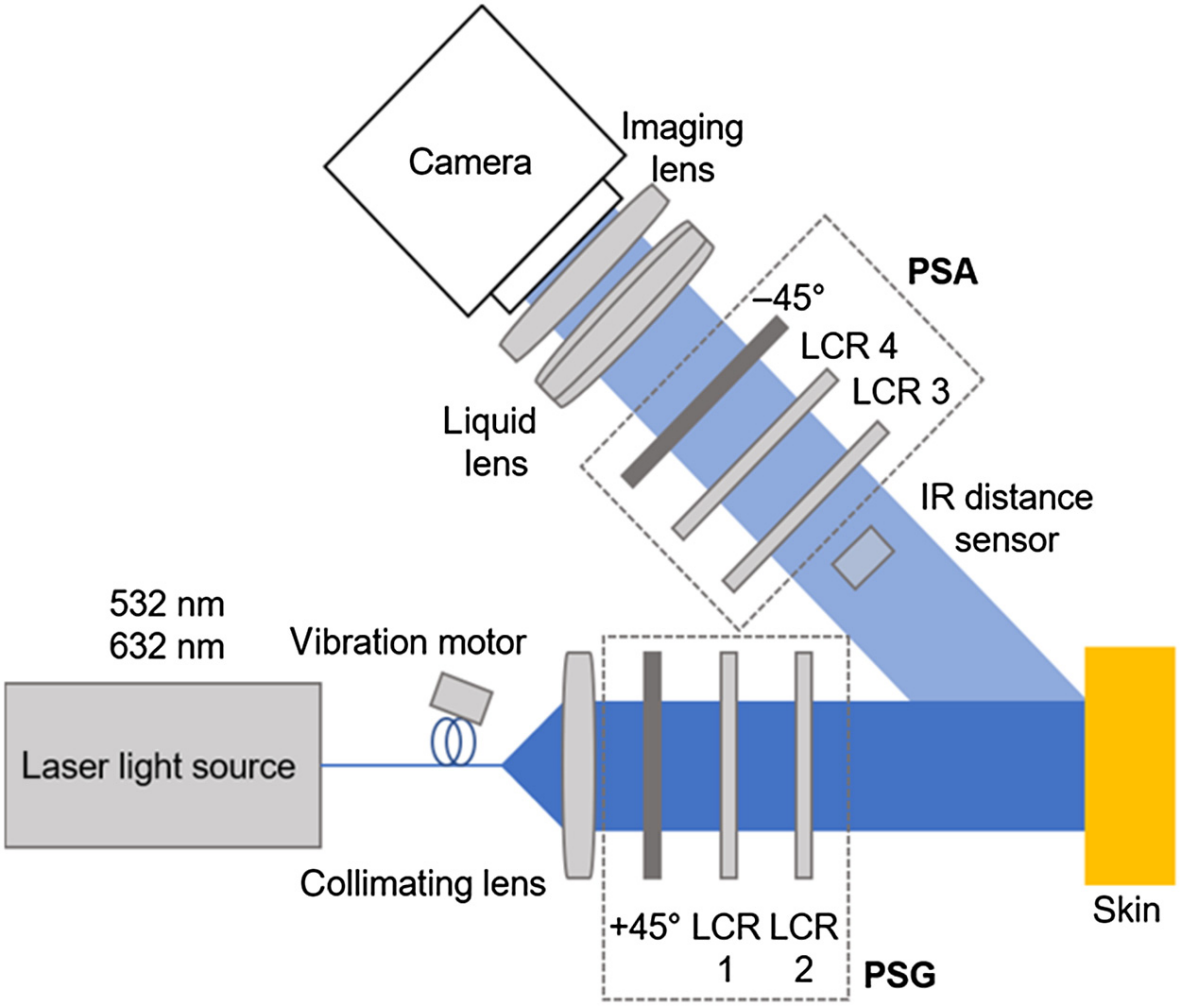
Sketch of the MM polarimeter used. The IR distance sensor for the automatic focus system is placed below the path of the beam. A continuous wave (cw) laser is coupled into an optical fiber with an attached vibrational motor for speckle reduction. Together with a fixed linear polarizer orientated at +45  deg with respect to the reference coordinate system, the liquid crystal retarders (LCR) 1 and 2 form the polarization state generator. The polarization state analyzer (PSA) contains two LCRs and a fixed linear polarizer orientated at −45  deg with respect to the reference coordinate system.

We use our in-house MM polarimeter^22^ for the measurements reported in this work. A sketch of the optical elements of the system is shown in Fig. 1.

**Fig. 2:**
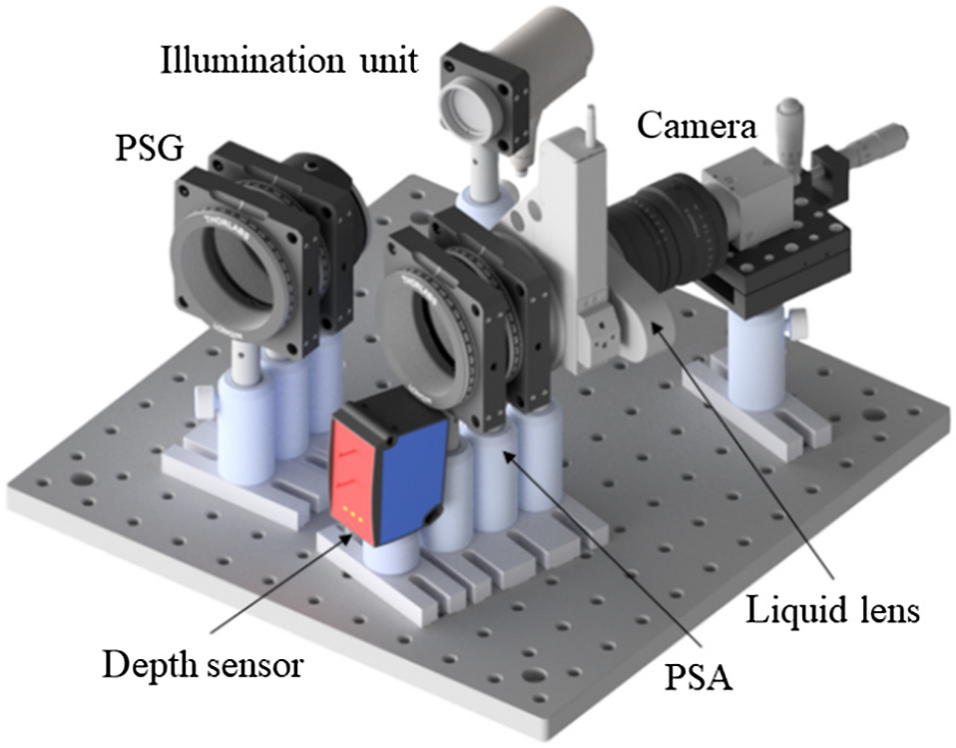
CAD design of autofocus system for a MM polarimeter.

The distance sensor (DT35-B15851, Sick AG, Waldkirch, Germany) measures the distance from a point close to (but outside) the polarimeter’s field of view to avoid interference with its illumination, as visualized in Fig. 2.

With the liquid lens, it is possible to adjust the focus in real-time based on the distance information provided by the distance sensor and on a function derived from the calibration procedure, which relies on the collection of value pairs of working distance and the liquid lens current. In detail, the liquid lens current is adjusted for different working distances until the imaged plane object is in maximum focus.^23^ The automatic focus works well for most skin parts within a theoretical working distance range of 30 cm to 8 m.^8^ However, the working distance is limited by the illumination intensity (and therefore noise) and the desired image resolution. With the automatic focus, it is possible to fully open the imaging aperture and accept the reduced depth of field, as larger openings reduce the acquisition time due to shorter possible exposure times.

**Fig. 3:**
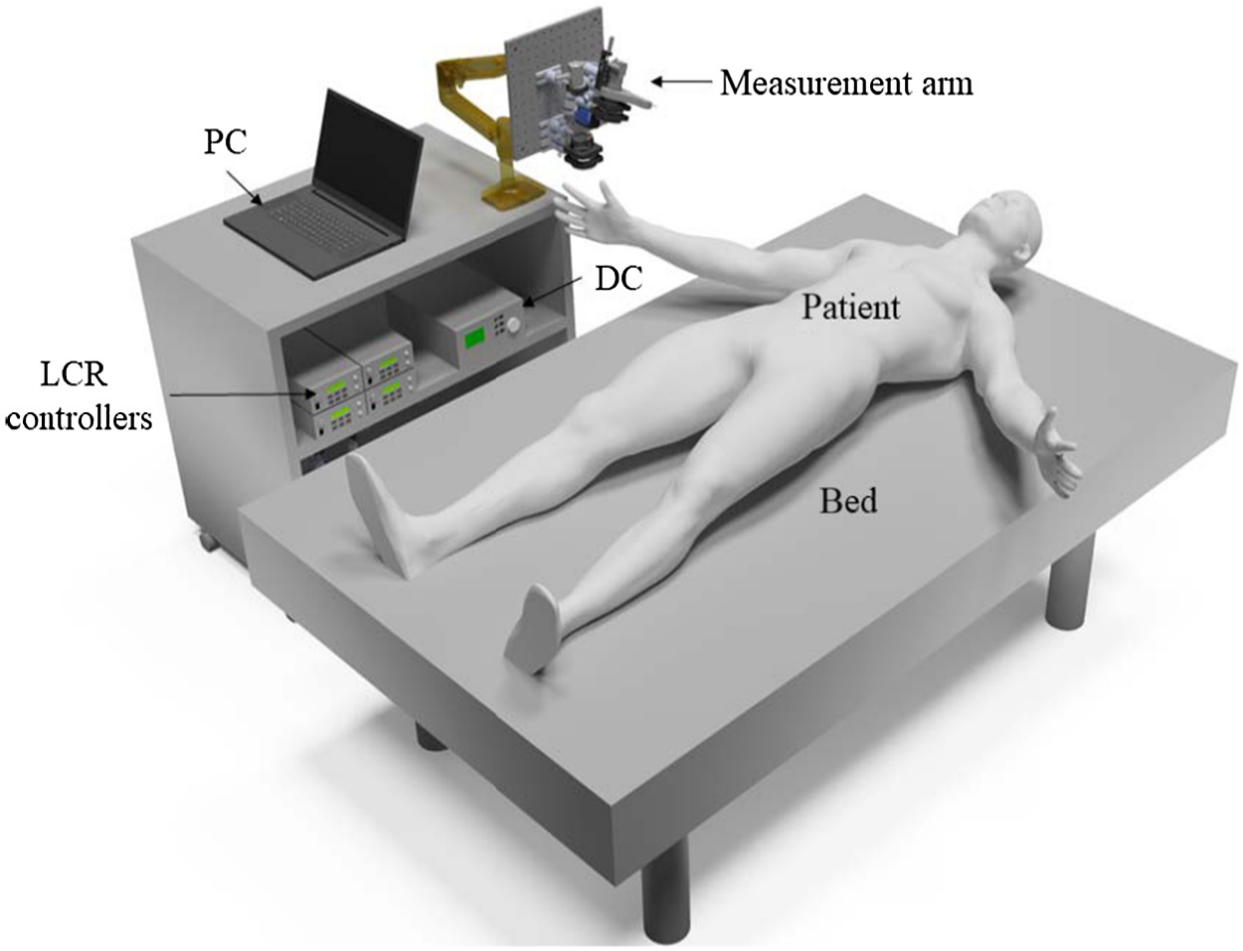
CAD design of the *in vivo* skin MMP imaging setup. The patient movement is minimized through the possibility of the patient lying down.

To minimize patient motion, we designed a mobile MM acquisition system based on an ergonomic arm (Ergotron LX ARM 45-241-026, Ergotron, St. Paul, Minnesota) that allows patients to lie on a patient bed. Figure 3 shows a CAD design of the complete measurement system.

The mobile MM acquisition system allows the operator to image essentially all parts of the human skin. For better visualization, the appropriate laser safety measures are not shown in this representation.

### 2.2 Image Processing

Polarimetric datasets are obtained with the MMP only under unintentional movement (e.g., breathing and shaking). In the following, we describe the different image registration techniques used in this work to correct for the misalignment caused by that motion. First, registration assessment is conducted on the basis of subjective criteria. In addition, an objective criterion is applied with the comparison of the numerical values of each polarimetric parameter, respectively.

### 2.3 Image Registration of Polarimetric Images

Image registration is the process of aligning two or more images of the same scene.^24^ It is often used as a precondition for other image processing applications.^25^ Determining an effective approach to image registration depends on the application case. Careful selection of a point transformation model is required to provide reference points between the images. In addition, a method for comparing information to identify the parameters necessary to correctly align images is needed. In general, there are two well-known methods for automatic image registration: feature-based and intensity-based registration algorithms.^26^ By contrast, manual image registration relies on control point mapping registration algorithms.^27^

Local features and their descriptors constitute the basis for many computer vision algorithms.^28^ Their applications include image registration,^29^ object detection^30^ and classification,^31^ tracking,^32^ motion estimation,^33^ and content-based image retrieval.^34^ Local features refer to a pattern or distinct structure that exists in an image, such as a point, edge, or small patch of the image. They are usually associated with an image patch that differs from its immediate environment in texture, color or intensity. Although the distinction from its environment is important, the representation of the content of the characteristic is usually not relevant. Examples of local features are blobs, corners, and edge pixels.^24^ Due to the lack of matching points in skin images, feature matching is not a reliable method for batch image processing alone.

The Image Processing Toolbox™ by MATLAB (MATLAB, 2021. version 9.11.0 (R2021b), Natick, Massachusetts: The MathWorks Inc.) provides tools for point mapping to determine the required transformation parameters to align images. In point mapping, the user selects points in a pair of images that identify the same reference. Then a geometric mapping is deduced from the positions of these control points.^24^ In selecting control points, a high level of accuracy is required. To align the target image, we need to select at least two pairs of matching points between the target image and the reference image. More pairs of matching points improve the registration result. This increases the processing time for batch image processing.

**Fig. 4:**
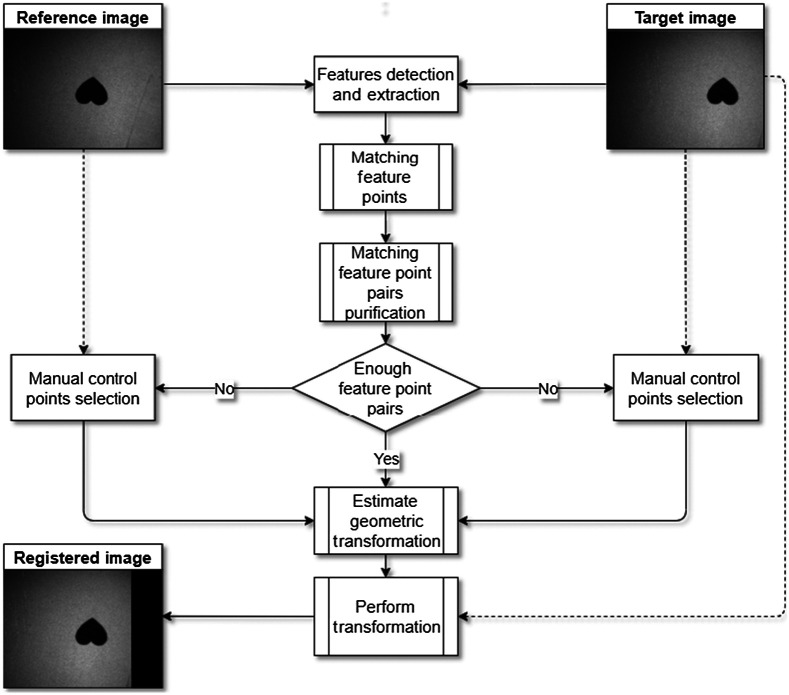
Flow chart of semiautomated keypoint-based registration with an example of a heart symbol printed on paper.

We propose a semiautomated key point-based registration method for polarimetric images to take into account the inevitable movement during acquisition. We combine the registration of both feature-based (automatic) and control point mapping (manual). Figure 4 shows a visual representation of this process.

The process starts with an algorithm based on KAZE^35^ features. In the case of insufficient pairs of matching points, it will switch to the manual mapping algorithm of the control points for this particular image. The Computer Vision Toolbox™ provides several methods for detecting corner and blob features and includes various descriptors. In this method, feature detection selects regions of an image with unique content, such as blobs. The feature detection finds possible points for subsequent processing that do not necessarily refer to physical structures. Finding features that remain locally invariant so that they are identifiable even in the presence of rotation or scaling is essential to feature detection.

Feature extraction involves the calculation of a descriptor, which is typically done on regions centered around detected features. By the means of image processing, descriptors transform a local pixel neighborhood into a compact vector representation. This new representation allows for comparison between neighborhoods, regardless of changes in scale or direction. We chose the best feature detector and descriptor from the perspective of our application criteria and the nature of our data by testing the available feature detector and descriptor combinations. Table 2 shows a comparison of the number of matching pairs between two images for different blob detectors and descriptors.

**Table 2:** Comparison of the number of matching pairs between two images for different blob detector and descriptor combinations. We exemplarily show the values for two images of the polarimetric raw data of a nevus (compare with Fig. 15) and a honey drop on healthy skin (compare with Fig. 13).

Blob detectors	Descriptor	Nevus			Honey drop on healthy skin		
		Features detected		Matching pairs between reference and target images	Features detected		Matching pairs between reference and target images
		Reference image	Target image		Reference image	Target image	
**KAZE**	**KAZE**	**9210**	**3668**	**1067**	**6616**	**3785**	**873**
	SIFT	10,994	4309	419	8456	4732	119
	SURF	9210	3668	484	6616	3785	346
	HOG	9206	3668	21	6612	3785	17
	BRISK	8497	3279	586	6142	3482	210
	FREAK	8925	3525	98	6430	3668	51
SIFT	SIFT	1388	482	55	633	139	8
	KAZE	1143	406	73	419	93	12
	SURF	1143	406	117	419	93	28
	HOG	1139	406	8	417	93	35
	BRISK	1101	380	56	408	89	13
	FREAK	1111	382	42	409	89	8
SURF	SURF	494	116	49	66	19	2
	KAZE	494	116	36	66	19	1
	SIFT	623	144	27	110	26	1
	HOG	494	116	67	66	19	0
	BRISK	452	105	36	62	16	0
	FREAK	489	111	22	64	18	2

Note: The detector and descriptor combinations employed in this article are highlighted in bold.

**Fig. 5:**
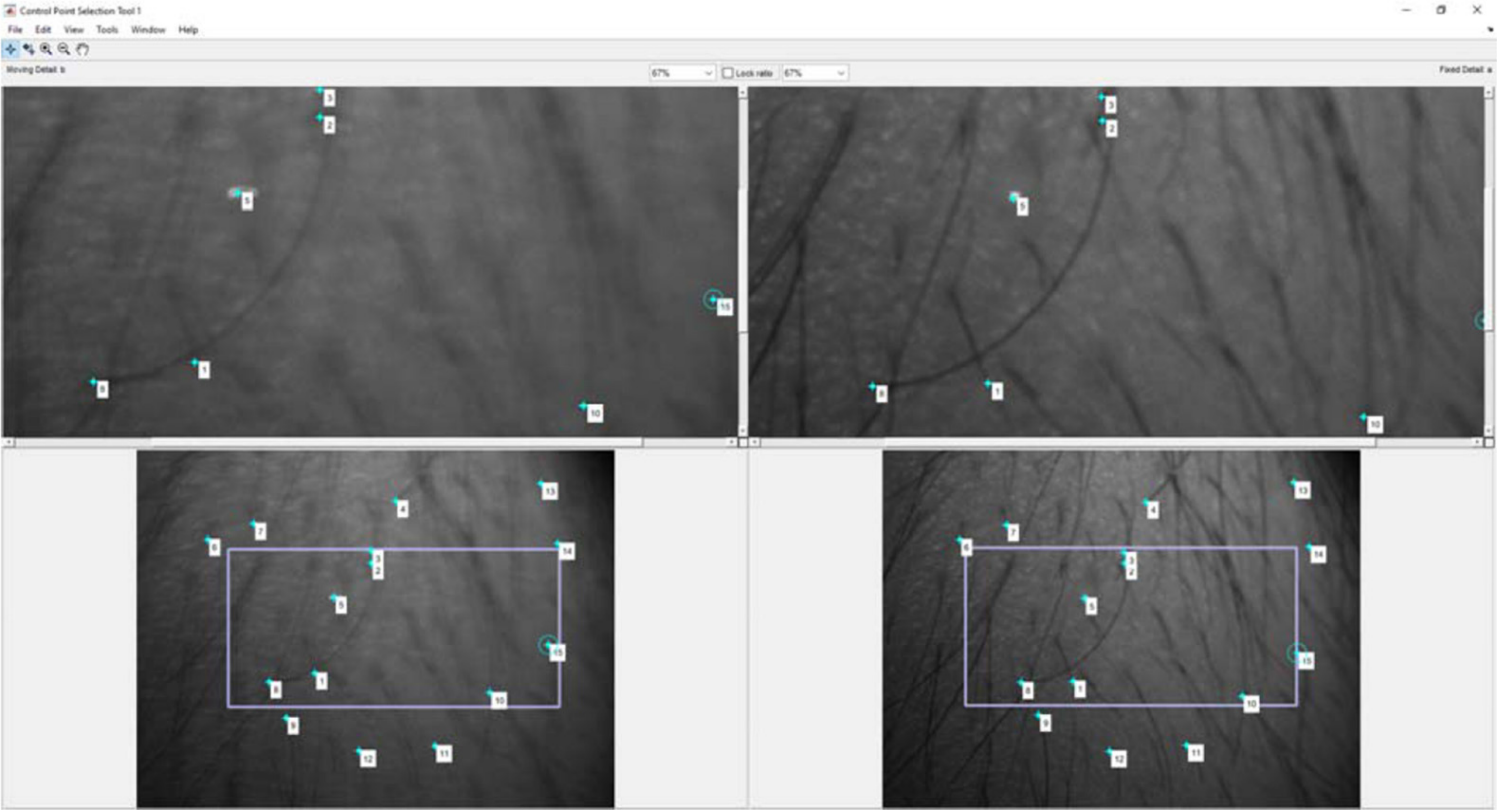
Screenshot of the manual control point selection process. The user selects the control points by clicking on a feature in the top-left image and then the corresponding feature in the top-right image. The lower row images are used for overview. The blue box region is shown enlarged in the upper row of images.

If there are sufficient valid matches, the false matches are removed by selecting only the strongest among them. This work is based on the M-estimator sample consensus (MSAC),^36^ a variant of the random sample consensus (RANSAC)^37^ algorithm. This method finds a geometric transform, separates the correct matches from the spurious matches, and then applies the computed geometric transform to the image being compared.^36^ For this method, a minimum of two matching pairs is required. If the requirement is not met, then it allows the operator to manually select the two matching pairs between the references and the particular target image. The manual selection of control points is shown in Fig. 5.

After alignment of all images, the MM is calculated. However, the edges of the spatially resolved MM entries are usually not valid and show distracting extreme values. To achieve results without distracting edges, we crop all images to the reference overlap zone of all aligned images or the area of interest.

## 3 Results

**Fig. 6:**
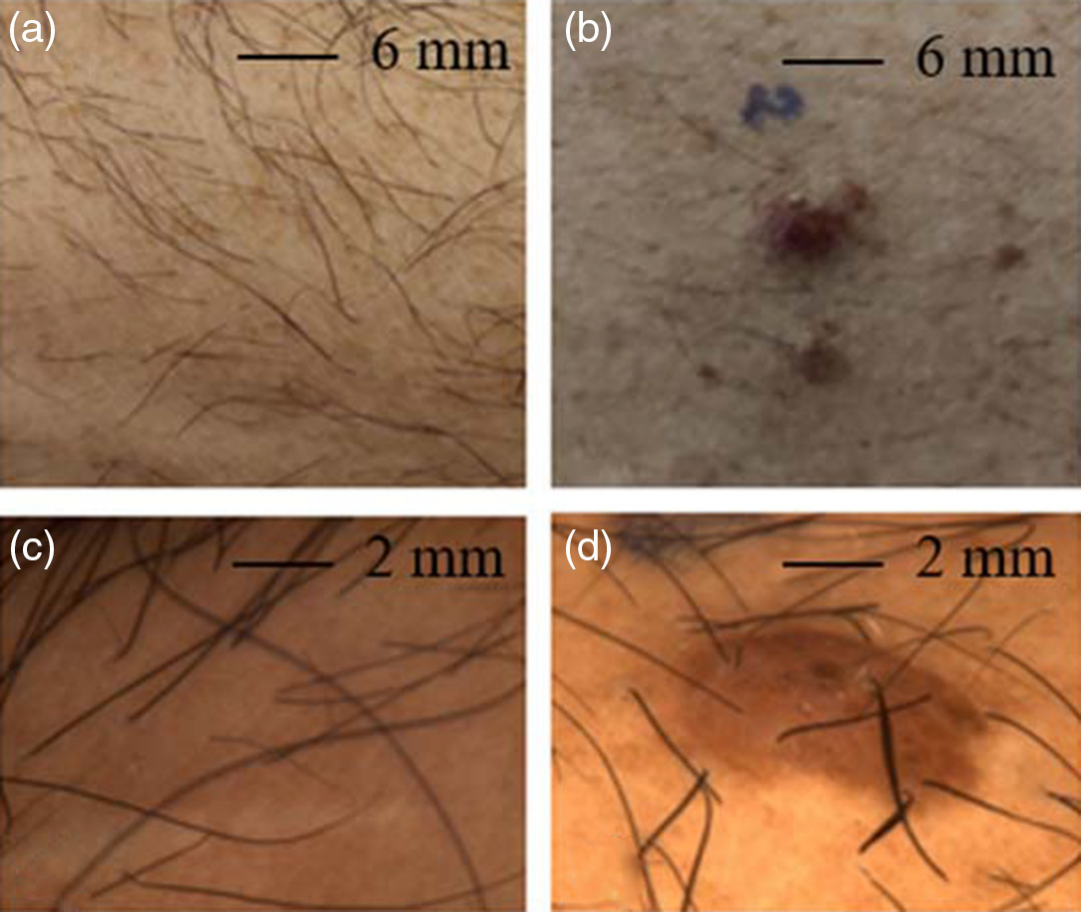
Clinical images of (a) healthy skin with hair and (b) a benign nevus. Dermoscopy images of (c) healthy skin with hair and (d) a benign nevus as a standard skin imaging reference to MM imaging.

Figure 6 shows the samples studied in a first step that were captured by a smartphone (upper row) and by a noncontact dermoscope^23^ (lower row).

We implement our approach on healthy skin with hair and a mole with surrounding skin, respectively. In addition, we apply a tiny drop of honey to the healthy skin as a phantom for small skin patches with varying optical activity.^38^ Aligning these samples is particularly important due to their strong contrast resulting from the hair and the nevus. The clinical and dermoscopic images serve as reference for the polarimetric skin imaging.

### 3.1 Registration of Polarimetric Data for *In Vivo* Skin Imaging

**Fig. 7:**
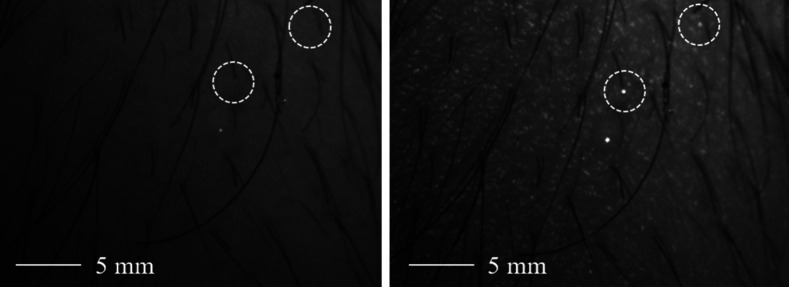
The visibility of features in human skin can depend on the polarization states. Two features with strong polarization dependency are marked exemplarily.

First, we show that in polarimetric data the visibility of features in the skin is usually polarization sensitive, as shown in Fig. 7.

From the comparison of the two images from the polarimetric raw data in Fig. 7, it is obvious that the visibility of features in human skin can depend on the combination of the state of the polarization state generator and the PSA.

**Fig. 8:**
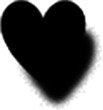
Phantom designed based on ABCD criteria.

In the following, we demonstrate that movement during data acquisition can lead to false MMs and derived polarimetric parameters. To do this, we use an icon as shown in Fig. 8 printed on paper as a lesion phantom.

**Fig. 9:**
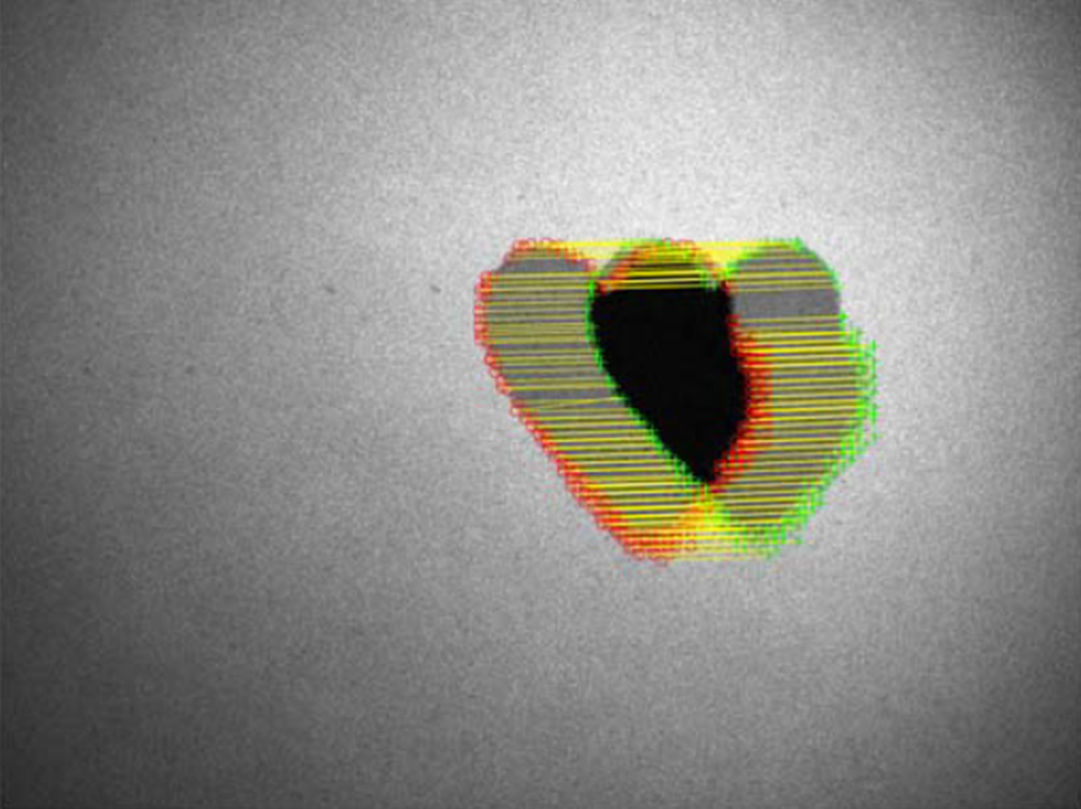
The detected matching features are at different positions in two exemplary overlayed raw images, as indicated by the yellow lines connecting corresponding matching points. This error of orientation is due to motion during the acquisition.

The phantom design is based on the ABCD criteria for melanoma diagnosis. It is asymmetric and has irregular borders and a diameter >5  mm. The phantom is moved throughout the acquisition. The translational and rotational movement within the focal plane, realized by manually moving the target on a rail with one additional rotational degree of freedom throughout the acquisition, leads to an error of alignment as shown in Fig. 9.

**Fig. 10:**
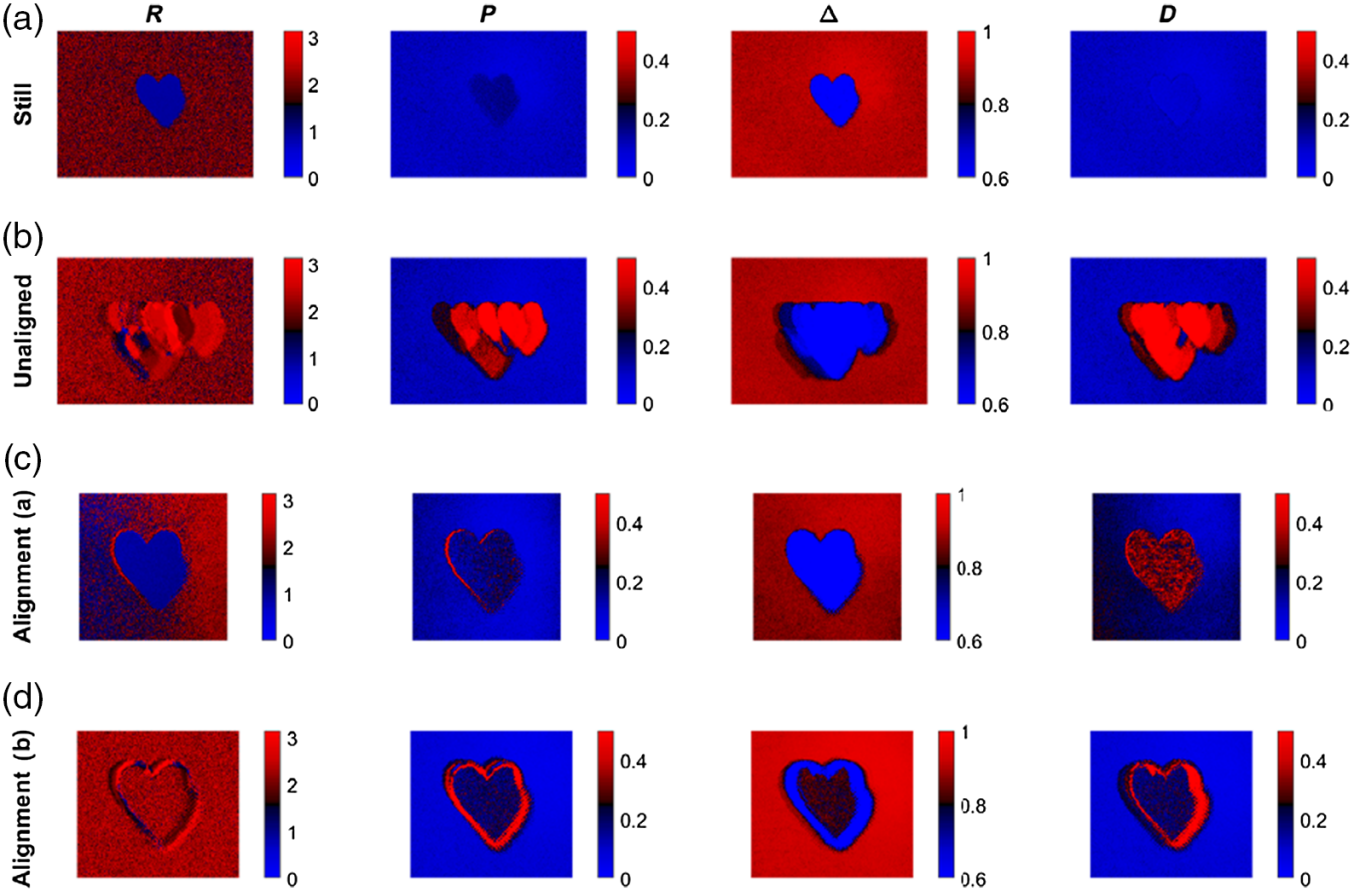
Polarimetric parameters of the phantom. (a) Not moved, (b) moved and unaligned, (c) feature-based alignment, and (d) intensity-based alignment.

In the following the spatially resolved polarimetric parameters retardance, polarizance, depolarization power, and diattenuation are calculated. In the remainder of this work, we refer to our feature-based registration method as alignment (a) and the standard intensity-based image registration method as alignment (b). As displayed in Fig. 10, the results differ largely depending on whether the polarimetric data were registered or not and which registration method was applied.

The previous comparison shows that registration has two immediate positive effects. First, image alignment leads to an improved image quality, as the features of the phantom are more clearly visible. Second, alignment corrects spatially resolved polarimetric parameters. The trend of retardance, polarization, and diattenuation of the phantom is reversed if the images are not aligned. The aligned and cropped data parameters indicate that the alignment has been achieved, but that the values on the phantom edges are still different compared with the static reference sample, as seen in the polarizance panels in Fig. 10, third and fourth rows. As shown in Fig. 10, it is also obvious that the polarimetric parameters from the raw data that have been registered with the feature-based method shows a greater agreement with the polarimetric data from the data at rest (ground truth) compared with the application of the standard intensity-based method. Further, the feature-based method is less prone to artifacts on the edges of the phantom.

As shown in Table 3, it is obvious that the feature-based registration leads to results that are closest to the ground truth for R, P, and Δ. For D, both alignment methods give a similar numerical value that does not match with the ground truth.

**Table 3:** Comparison of the numerical values of the determined polarimetric parameters from the center of the phantom.

	R		P		Δ		D	
	Mean	Std	Mean	std	Mean	std	Mean	std
Still	0.8680	0.0230	0.1571	0.0224	0.4719	0.0625	0.0890	0.0146
Unaligned	2.2888	0.5104	0.5034	0.1852	−0.1163	0.9973	0.6074	0.2471
Alignment (a)	**0.9077**	0.0988	**0.1820**	0.0599	**0.4314**	0.0763	0.3103	0.0726
Alignment (b)	2.1826	0.4073	0.2567	0.1956	0.5574	0.6153	0.3071	0.2743

Note: Significant values are highlighted in bold.

**Fig. 11:**
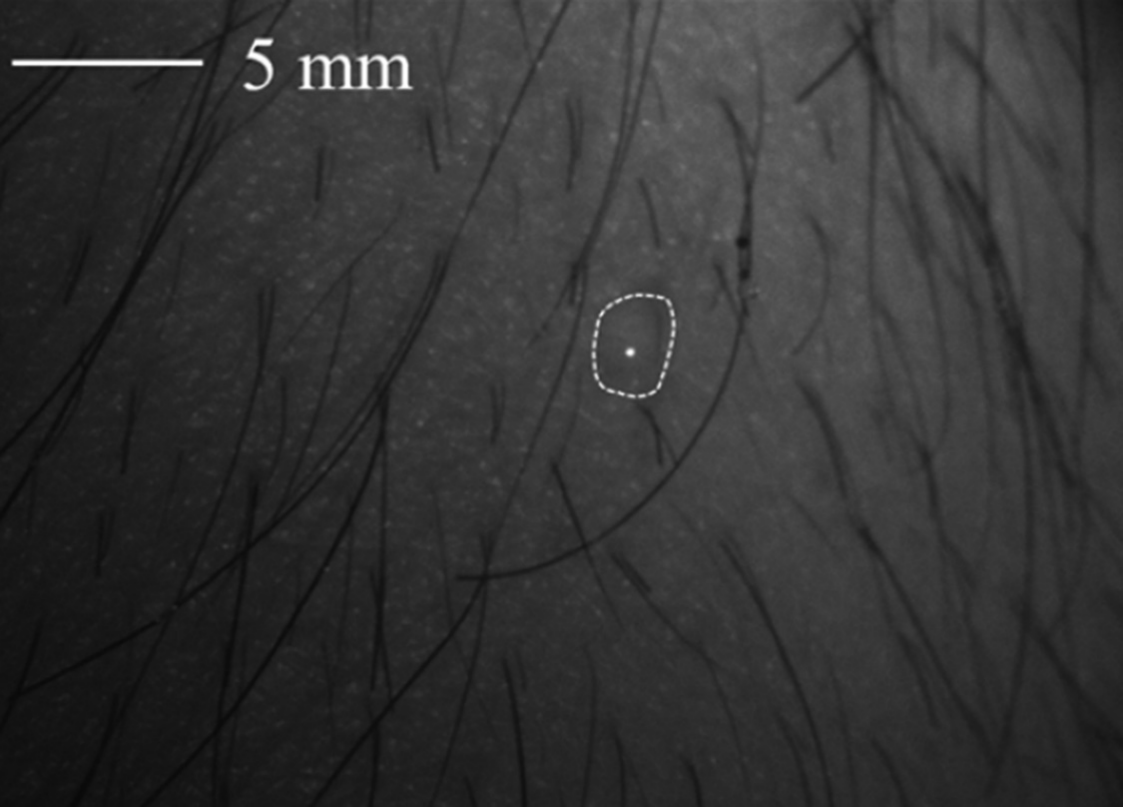
Exemplary raw data of the honey drop on healthy skin as a phantom for spatially defined small changes in polarization on human skin. The contours of the honey drop are marked.

Next, a polarimetric *in vivo* measurement of a honey drop on healthy skin of a human arm with hair, as shown in Fig. 11, is carried out.

**Fig. 12:**
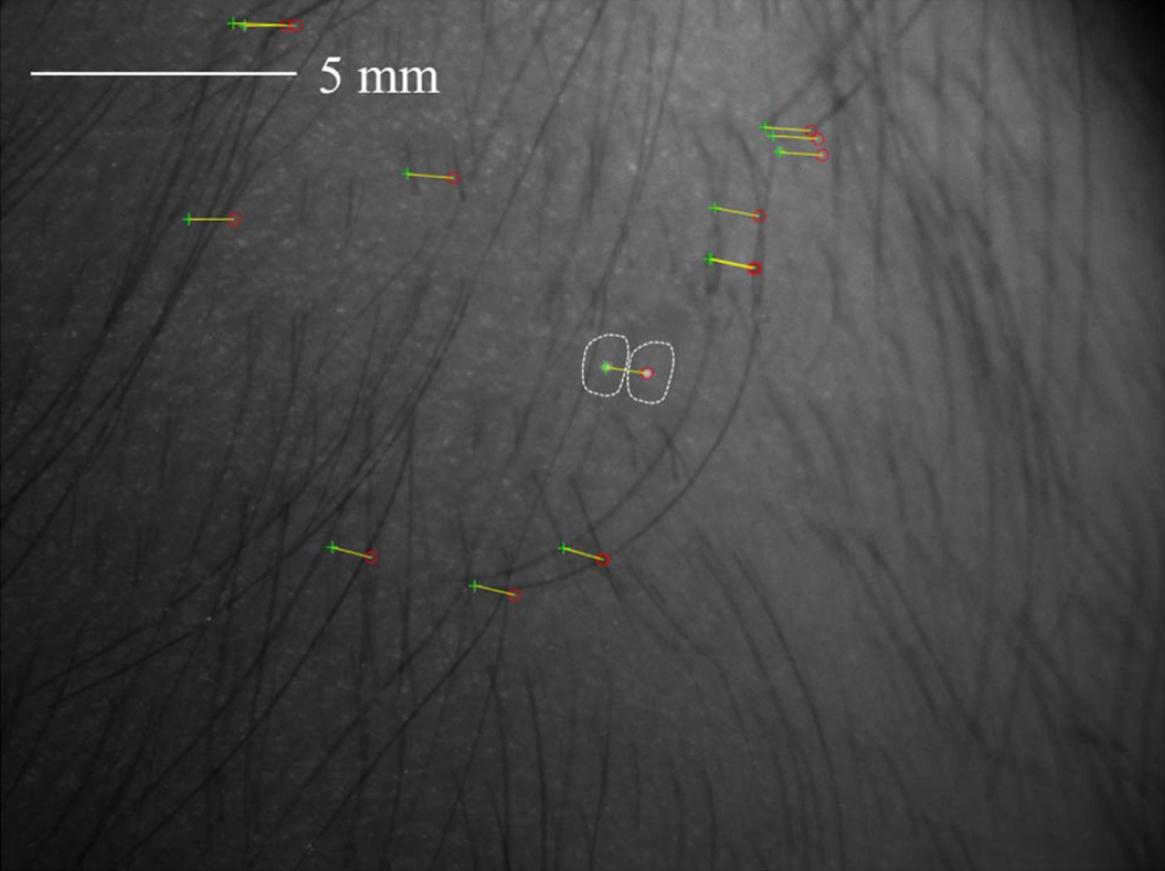
Exemplary resulting misalignment of the honey drop on healthy skin due to motion during the acquisition. Two exemplary images of the raw data are overlayed. The yellow arrows connect corresponding matching points. The region of the honey drop is marked in both images.

The diameter of the honey drop is ∼2  mm. Figure 12 shows the degree of unintentional motion during data collection by comparing the position of detected features in two images of the polarimetric data.

**Fig. 13:**
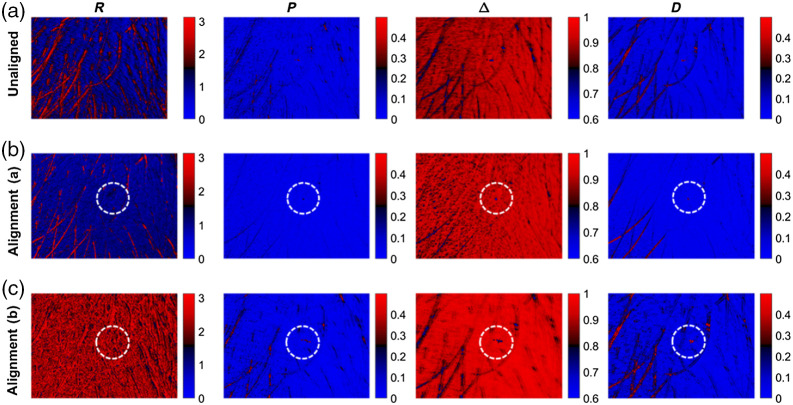
Polarimetric parameters for a drop of honey on healthy skin with hair. (a) unaligned, (b) feature-based alignment, and (c) intensity-based alignment. The region of the honey drop is marked. The contours are best identified in the case of the feature-based alignment due to the greatest contrast.

Figure 12 shows the degree of unintentional motion and the need for registration of the polarimetric data. The spatially resolved polarimetric parameters for a drop of honey on healthy skin with hair are displayed in Fig. 13 for the raw data as well as after feature-based and intensity-based alignment.

Feature-based registration of polarimetric images has an immediate effect on spatially resolved polarimetric parameters. After registration, the hair can be distinguished from the skin, and the range of values has changed. In addition, the tiny drop of honey is most distinguishable from the surrounding skin in the case of feature-based registration, especially in the Δ-patch, showing that such polarimetric changes in small skin patches can only be restored with a suitable image registration method.

**Fig. 14:**
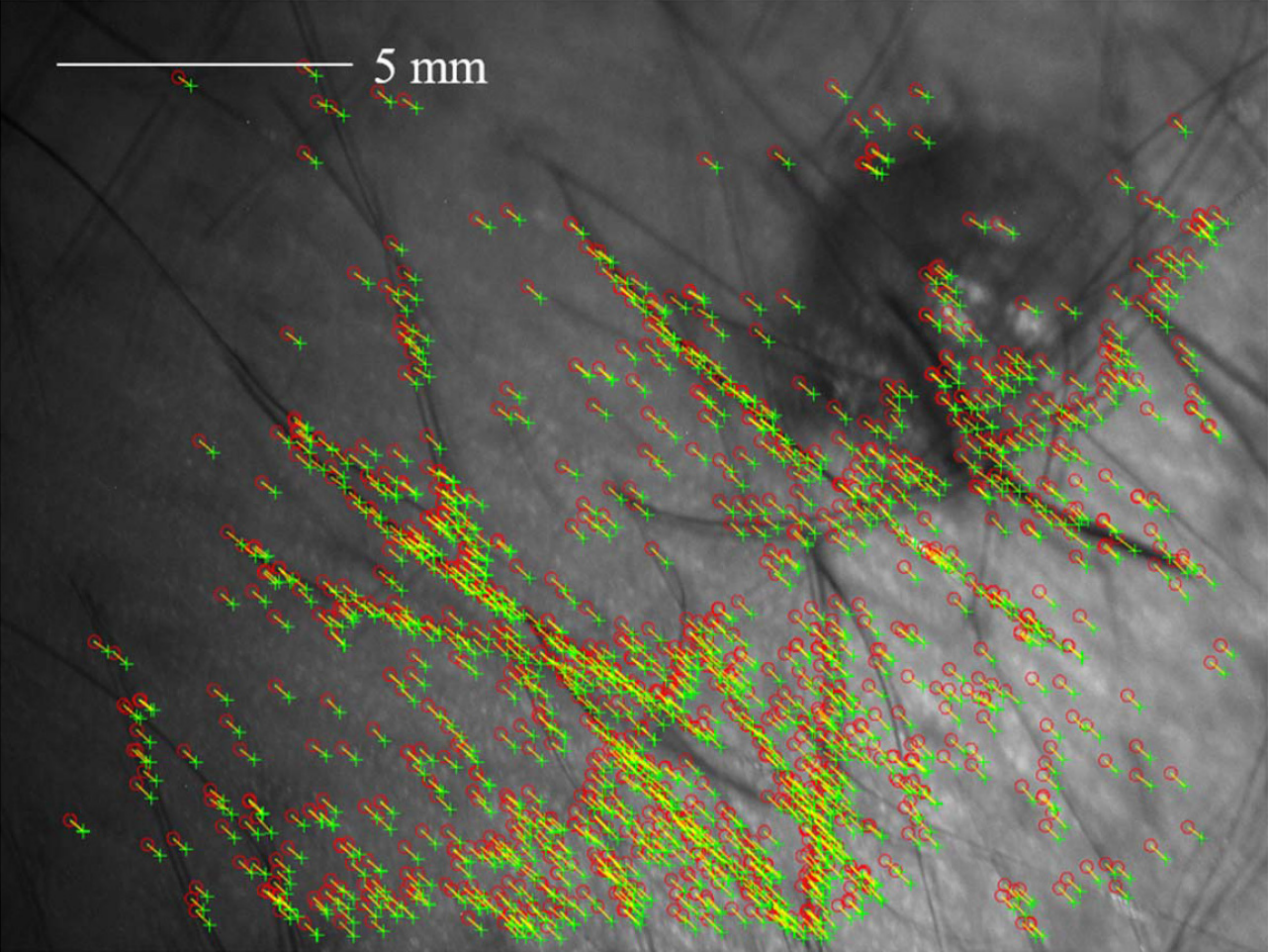
Exemplary resulting misalignment of matching feature points of the nevus due to motion during the acquisition. Two exemplary images of the raw data are overlayed. The yellow arrows connect corresponding matching points.

Subsequently, the influence of in-focal plane motion on the polarimetric parameters for *in vivo* nevus assessment was investigated. An *in vivo* measurement with our MMP is performed on a benign nevus of a volunteer. The degree of misalignment is obvious from Fig. 14, as the matching feature points are detected at slightly different positions.

**Fig. 15:**
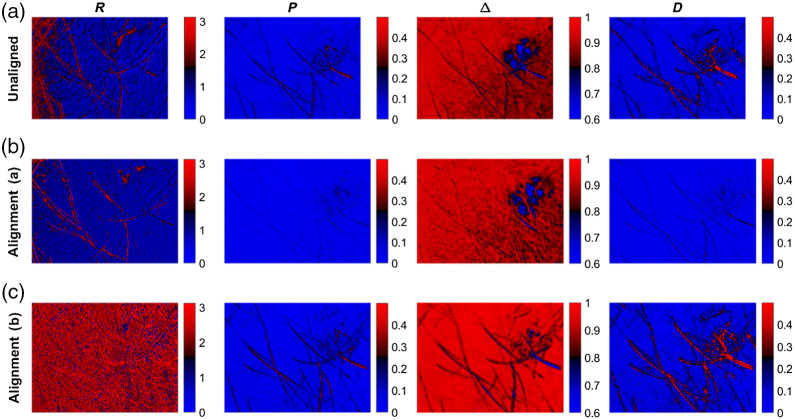
Polarimetric parameters for a mole. (a) unaligned, (b) feature-based alignment, and (c) intensity-based alignment.

It is clear from Fig. 14 that the features of the nevus do not overlap in the polarimetric images due to motion and that the polarimetric data must therefore be registered. In Fig. 15, the polarimetric parameters of a nevus of unaligned and aligned data are shown.

The feature-based registration of polarimetric data allows for the *in vivo* assessment of nevi. By contrast, unaligned or intensity-based aligned polarimetric data lead to artifacts in the polarimetric parameters that interfere with dermatological interpretation. The inconsistencies of polarimetric parameters at the edge of the nevus, as obvious from the retardance patch in the second row in Fig. 15, suggest that the image registration can be further improved. It is necessary to examine, in future work, whether the inconsistencies at the edges of the nevus are caused by the occurrence of biological and structural changes or by a slight residual misalignment.

### 3.2 Autofocus for Polarimetric *In Vivo* Skin Imaging

**Fig. 16:**
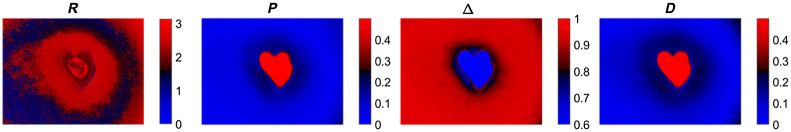
Polarimetric parameters from data of the phantom with motion in the direction of the optical axis without autofocus. The induced artifacts are obvious in comparison with the phantom at rest in Fig. 9, first row.

Finally, we investigated the effect of movement in the direction of the optical axis of the polarimetric system and the associated changes in the polarimetric parameters. For this, the focal plane was changed during the acquisition of the polarimetric parameters displayed in Fig. 16.

In comparison with Fig. 10, first row, it is clear that the change of the focus plane during acquisition leads to a bad focus on some of the images of the polarimetric data, which results in artifacts on the edges of the phantom. Consequently, an automatic focus system is required to take into account the skin movement in the direction of the optical axis during acquisition.

## 4 Conclusions

We implemented a semiautomatic feature-based registration method and studied the impact of different registration methods on polarimetric analysis. In addition, we found mapping functions that are suitable to reliably detecting the corresponding skin features as key points in polarimetric data. The body movement can induce motion blur because of relatively high exposure times. Furthermore, the movement of the skin in the 36 images acquired leads to false polarimetric parameters, preventing reliable diagnosis. In rare cases, some key points leave the field of view due to movement. To avoid false MM results, the characteristics of the 36 images are aligned using image processing techniques. In addition, the MMP is mounted on a movable arm that enables the system to be placed easily in the skin area studied while the patient movement is minimized with the patient bed setting. The results indicate that our methodology is a good solution to the problem. Further research into the feature detection technique is needed to enable a fully automated and reliable registration of *in vivo* skin polarimetry data. The results emphasize the importance of adequate image registration techniques for *in vivo* skin polarimetry. Thus, equipped with image registration, MM polarimetry could be a valuable asset for dermatology. The performance of image registration is most important when the skin area of interest is particularly small, as characteristics of polarimetric activity can be lost without data registration. Our proposed method is suitable for the alignment of polarimetric images overcoming the problems of intensity and threshold matching. In the future, we will use the approach for *in vivo* measurements of inflammatory skin diseases and melanoma skin cancer.

## Code, Data, and Materials Availability

Data are available upon reasonable request.
